# Low Employment Precariousness as a Structural Determinant of Nurses’ Well‐Being: The Mediating Role of Psychosocial Working Conditions

**DOI:** 10.1155/jonm/7165363

**Published:** 2026-10-31

**Authors:** Marja Hult, Venla Karikumpu, Marjo Ring, Santtu Mikkonen

**Affiliations:** ^1^ Department of Nursing Science University of Turku Kiinamyllynkatu 10 20520 Turku Finland utu.fi; ^2^ Department of Sustainable Well-Being South-Eastern Finland University of Applied Sciences Patteristonkatu 3 50100 Mikkeli Finland; ^3^ Department of Social Sciences University of Eastern Finland Yliopistonranta 8 70210 Kuopio Finland uef.fi; ^4^ Department of Environmental and Biological Sciences University of Eastern Finland Yliopistonranta 8 70210 Kuopio Finland uef.fi

**Keywords:** burnout, employment precariousness, mental well-being, nursing, psychosocial working conditions

## Abstract

**Background:**

Nurses experience high levels of impaired mental well‐being and burnout, threatening both workforce sustainability and the quality of care. Despite growing recognition of employment precariousness as a structural determinant of health, limited attention has been paid to its role in shaping nurses’ workplace experiences. Although nursing is generally regarded as a secure profession, considerable variation in employment conditions exists within the workforce, contributing to experiences of employment precariousness. However, the mechanisms linking employment precariousness to nurses’ well‐being remain insufficiently understood.

**Aim:**

To examine the associations of low employment precariousness on mental well‐being and burnout among nurses and to analyse how psychosocial working conditions mediate these associations.

**Methods:**

This prospective longitudinal study collected survey data from nurses in 2023 and 2025, including primarily registered nurses (66%) and practical nurses (19%). A total of 1018 nurses participated in both survey waves. Low employment precariousness was measured across eight dimensions. Psychosocial working conditions were assessed through job resources, psychological safety, social support and work engagement. Path analysis models were used to examine the longitudinal effects of low employment precariousness and to assess the mediating role of psychosocial working conditions.

**Results:**

Low employment precariousness is associated with better mental well‐being and lower burnout. It also showed positive cross‐sectional and cross‐lagged effects on mediating factors, which, in turn, associated with mental well‐being and burnout. Low employment precariousness in 2023 had an indirect positive effect on mental well‐being in 2025 (*β* = 0.20) and an indirect negative effect on burnout in 2025 (*β* = −0.19).

**Conclusion:**

This study provides novel evidence that low employment precariousness may have long‐term beneficial effects on nurses’ well‐being. Psychosocial resources mediate the relationship between low employment precariousness and well‐being at both the organizational and individual levels. The findings underscore the importance of fair and inclusive management as a key focus for workplace interventions in healthcare settings.

## 1. Introduction

While many interventions addressing workforce shortages and stressors in healthcare have focused on individual‐level solutions [1], structural and contextual dimensions remain underexplored. Limited attention has been paid to evolving employment dynamics and systemic capacity to ensure secure, predictable and ethically sustainable working conditions [2]. Globalization, liberalization and the growing demand for workforce flexibility have intensified the employment precariousness [3]. In the care sector, these forces, combined with privatization and commercialization, particularly in elderly and long‐term care, have contributed to the rise of employment precariousness [4], which is characterized by job insecurity, lower wages and limited access to social benefits and legal protections [5]. Workers in precarious jobs often experience cumulative disadvantages, including weak labour law protections, reduced union representation and poor enforcement of standards [6]. These jobs frequently lack benefits, such as health insurance, paid leave, pensions and unemployment coverage, and are often associated with hazardous conditions, long hours and inadequate safety measures, all factors that heighten the risk of occupational injuries and stress [5].

Employment precariousness in nursing refers to unstable and insecure working arrangements, such as temporary contracts, part‐time or agency work, unpredictable schedules, low wages and limited employment benefits [4, 7, 8]. These conditions have become increasingly common due to outsourcing, cost‐containment measures and flexible staffing models in healthcare systems. Evidence shows that employment precariousness negatively affects nurses’ well‐being by increasing exposure to job insecurity, work overload and poor psychosocial working conditions [8]. Nurses working under precarious conditions report higher levels of burnout, depression, anxiety, psychological distress, absenteeism and presenteeism, as well as lower job satisfaction and stronger intentions to leave the profession [9–11]. These relationships are partly explained by psychological mechanisms such as moral distress and work–family imbalance, while social support, psychological capital and resilience can buffer some of the negative effects [8, 12]. Young nurses, women and migrant nurses are particularly vulnerable to precarious employment conditions, which can reduce access to workplace resources and support [4, 13]. Overall, employment precariousness has been identified as a significant threat to both workforce sustainability and the quality of working life in nursing [8, 14].

Notably, most research on employment precariousness, including the studies cited above, examines only one or a few dimensions of the phenomenon. However, focussing on a single dimension, most commonly job insecurity, fails to capture the full complexity of employment precariousness [5]. Therefore, the use of multidimensional measures that capture the cumulative disadvantages of precarious employment is needed to provide a more comprehensive understanding of its impacts on workers’ health and well‐being [6]. Moreover, to inform healthcare management, policymakers and labour market stakeholders, it is crucial to examine how low employment precariousness, that is, high‐quality employment as opposed to precarious employment, affects nurses’ well‐being over time. Existing research has linked employment precariousness to poorer well‐being among nurses; however, the evidence is based predominantly on cross‐sectional studies, highlighting the need for longitudinal research that can establish whether these associations persist over time. Furthermore, greater understanding is needed of the mechanisms through which employment conditions influence nurses’ well‐being in workplace settings. Therefore, studies examining the psychosocial working conditions that mediate the relationship between low employment precariousness and nurses’ mental well‐being are warranted.

Job resources, as defined within the job demands–resources (JD‐R) theory, refer to positive aspects of work that facilitate the achievement of job goals, reduce job demands, or promote personal growth and development. These resources may be physical, psychological, social, or organizational in nature and include elements such as skill variety, autonomy, and performance feedback [15]. They can be conceptualized at multiple levels: interactional and relationship‐based resources at the group level, leadership‐related resources at the leader level, and organizational resources such as role clarity and opportunities for development [16]. Job resources have consistently been shown to enhance work engagement [17], which is considered a key component of occupational well‐being. Furthermore, organizational support through job resources has been associated with reductions in burnout among healthcare workers [18].

Psychological safety in healthcare is understood as a group‐level phenomenon embedded in a nonpunitive organizational culture that encourages interpersonal risk‐taking and fosters strong interpersonal relationships [19]. A positive psychosocial safety climate predicts healthcare workers’ psychological well‐being, whereas its absence increases psychological distress, emotional exhaustion, and workplace bullying [20]. Psychological safety has also been shown to mitigate burnout among healthcare professionals [21]. Workplace trust, ethical leadership, and relational leadership further reinforce psychological safety, contributing to overall well‐being [22].

Social support is defined as the provision of resources through social relationships to meet individual needs, taking into account the characteristics of both provider and recipient [23]. A systematic review emphasizes the role of social support as a coping strategy for enhancing healthcare workers’ mental well‐being [24]. Support from leaders and peers was particularly critical during the pandemic. Social support also serves as a protective factor against burnout [25], facilitated by positive leadership practices such as ethical leadership. Expressions of being valued by peers and reciprocal support for professional development have been linked to improved well‐being [26].

Work engagement, originally defined as the allocation of personal resources toward physical, emotional and cognitive aspects of work, is often considered the opposite of burnout [27]. Adequate working time, manageable workload and respect for professional contributions further enhance work engagement and reduce burnout. Job autonomy, including self‐leadership, also positively influences work engagement and overall well‐being [28]. Finally, decent working conditions, i.e., high‐quality work, have been shown to foster work engagement [29].

This study examines low employment precariousness as a structural determinant of nurses’ psychosocial working conditions, burnout and mental well‐being using a longitudinal design. Specifically, we investigate whether psychosocial working conditions mediate the associations between low employment precariousness and subsequent burnout and mental well‐being. The hypotheses are as follows:

 H1: Low employment precariousness associates positively with mental well‐being and negatively with burnout.
 H2: The effects of low employment precariousness on mental well‐being and burnout are mediated by psychosocial working conditions (see Figure 1).

**FIGURE 1:**
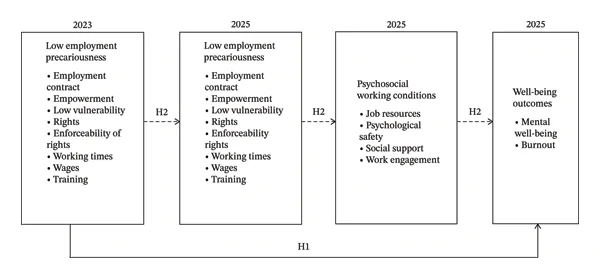
Study design with hypotheses.

## 2. Materials and Methods

### 2.1. Study Setting and Recruitment

This prospective longitudinal study was conducted in Finland with two data collections, T1 in February 2023 and T2 in February 2025. The biggest trade union representing public, private and third‐sector healthcare workers published an invitation for participating in the study in their weekly newsletter. Approximately 100,000 workers received the newsletter. A responding to the self‐administered questionnaire was done online by Webropol tool. An invitation to the study was reminded twice. A total of 4574 nurses responded to the survey in 2023 (T1). At the end of the survey, respondents were invited to provide their email address if they were willing to participate in the follow‐up survey. In 2025, the follow‐up survey was emailed to those who had volunteered to participate (*n* = 1796), and 1018 individuals responded. Of these participants in T2, eight were excluded as there were over 50% of missing values giving a final sample size of 1010 (Figure 2). Dropout analyses revealed significant differences in gender, work experience and educational level. Compared with non‐dropouts, dropouts were less often men (6.2% vs. 8.2%), had longer work experience (mean 17.1 [SD 11.3] vs. 15.9 [SD 10.2] years), and were less likely to hold a master’s degree or higher (9.3% vs. 12.7%). Thus, dropouts were more often women, had longer work experience, and lower educational attainment than participants who completed the follow‐up survey.

**FIGURE 2:**
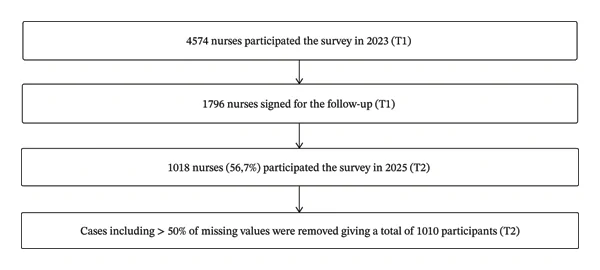
Flow chart for the study participation.

### 2.2. Data Collection

#### 2.2.1. Low Employment Precariousness

The Employment Precariousness Scale (EPRES‐BE) [6] was used, with items reverse‐coded, to measure low employment precariousness. The scale included 39 items across eight dimensions: (1) employment contract (permanent employment contract; 1 item), (2) empowerment (formal and informal empowerment and participation; 4 items), (3) low vulnerability (low authoritarianism, access to information and procedural justice; 11 items), (4) rights (workers’ rights; 4 items), (5) enforceability of rights (ability to exercise rights; 6 items), (6) working times (predictability of working hours, low levels of unsocial and flexible working hours and a low amount of long hours/overwork; 10 items), (7) wages (monthly net income; 1 item) and (8) training (employer‐provided training; 1 item). Cronbach’s alpha values were 0.879 in 2023 and 0.873 in 2025, indicating high internal consistency. For dimensions comprising multiple items, mean scores were calculated and harmonized to a scale ranging from 0 to 1, with higher values indicating higher levels of low employment precariousness.

#### 2.2.2. Outcomes

*Mental well-being* was measured with Mental Well‐Being Index (WHO‐5) with five items [30], an example being “I have felt calm and relaxed.” Response options ranged from 1 (*never*) to 6 (*all the time*), and a mean was calculated from all the items. A higher mean indicated better mental well‐being. Cronbach’s alphas were 0.879 in 2023 and 0.894 in 2025.

*Burnout* was assessed with the Burnout Assessment Tool (BAT‐4) instrument [31], and translated and validated in Finnish [32]. Four‐item scale captured four fundamental dimensions of burnout: exhaustion, mental detachment from work, cognitive impairment and emotional impairment. Responses were provided on a five‐point Likert scale from 1 (*never*) to 5 (*always*). A mean was calculated for all the items, and a higher mean indicated higher burnout. Cronbach’s alpha was 0.742 in 2023 and 0.701 in 2025.

#### 2.2.3. Mediators

*Job resources* are factors that can foster engagement and buffer the impact of job demands [15]. This study included the following job resources: the possibility and requirement for learning, professional competence, creativity, task variation, voice and possibility in decision‐making concerning own work. The items were answered with the options from 4 (*strongly agree*) to 1 (*strongly disagree*). Mean of all the items was calculated, and a higher value indicated better job resources. The internal consistencies were acceptable, as *α* = 0.751 in 2023 and *α* = 0.759 in 2025.

*Psychological safety* was measured with four items derived from the Psychological Safety Scale [33]. These items were designed to assess perceptions of psychological safety within the work environment. They included statements such as *In this work environment, I find it difficult to speak up if I notice problems in patient care* (reverse‐coded) or *Staff find it easy to ask questions if they do not understand something.* Participants indicated their level of agreement using a Likert scale ranging from 5 (*completely agree*) to 1 (*completely disagree*). Higher scores reflected a higher perceived level of psychological safety. Cronbach’s alphas were 0.692 in 2023 and 0.661 in 2025.

*Social support* was assessed using seven items, with three addressing support from colleagues and four focussing on support from managers. All items referred to experiences during the preceding week. Example statements include *My colleagues help me to do the work* and *My manager cares about my well-being.* Responses were collected on a four‐point Likert scale ranging from 4 (*totally agree*) to 1 (*totally disagree*), where higher scores reflected stronger perceived social support. A mean score was computed across all items. The scale demonstrated good internal reliability, with a Cronbach’s alpha of 0.841 in 2023 and 0.844 in 2025.

*Work engagement* was measured using the Finnish adaptation of the nine‐item Utrecht Work Engagement Scale (UWES‐9), originally derived from the 17‐item version developed by Schaufeli et al. [34]. The instrument comprises three subscales—vigour, dedication and absorption—each represented by three items. An example item is *When I get up in the morning, I feel like going to work.* Responses were recorded on a seven‐point Likert scale ranging from 6 (*daily*) to 0 (*never*), with higher scores reflecting greater levels of work engagement. The scale demonstrated excellent internal consistency in this study, with a Cronbach’s alpha of 0.951 in 2023 and 0.950 in 2025.

### 2.3. Data Analysis

Cases with more than 50% of missing data were removed (*n* = 8). There were missing values from 0 to 2.7% by study variables. Little’s MCAR test showed that data were missing completely at random (*p* = 0.645); therefore, we used listwise deletion of missing values. Descriptive statistics as means, standard deviations and frequencies were used to describe the study participants. Internal consistencies of scales were calculated as Cronbach’s alphas. Correlations between the low employment precariousness dimensions by year, as well as between all the study variables, were calculated as Pearson’s correlation estimates and interpreted as very strong when *r* ≥ 0.80, strong when *r* ≥ 0.50, moderate when *r* ≥ 0.30 and weak if *r* < 0.30. Repeated‐measures ANOVAs were performed to examine changes over time. Partial eta squared (*η* *p* ^2^) was used as a measure of effect size. Effects were interpreted small when *η* *p* ^2^ > 0.03, medium when *η* *p* ^2^ > 0.08 and large when *η* *p* ^2^ > 0.18.

Confirmatory factor analyses (CFAs) were run by year to test the eight‐factor structure, i.e., eight dimensions, of low employment precariousness. Path models were used to test the hypotheses [35]. The following fit indices were applied to assess a model fit: root mean square error of approximation (RMSEA) < 0.05, Comparative Fit Index (CFI) and Tucker–Lewis index (TLI) > 0.95 indicated a good model fit [36]. For comparisons between nested models, chi‐square difference tests (Δχ^2^) were conducted. Changes in CFI (ΔCFI), TLI (ΔTLI) and RMSEA (ΔRMSEA) were examined to evaluate whether the inclusion of additional parameters improved model fit. Respecifications were guided by modification indices and theoretical plausibility. The significance was set at the level of *p* ≤ 0.05. The analyses were performed with Stata Version 19.5.

### 2.4. Ethical Considerations

This study was performed in line with the principles of the Declaration of Helsinki. According to Finnish legislation, this type of study does not need ethical approval. The trade union granted research permission, and participants provided informed consent before completing the survey.

## 3. Results

The mean age was 46.3 years (SD 10.2), and they had 15.8 years of work experience in average (SD 10.3). The majority were women (*n* = 919), 79 were men, and 8 were other or did not prefer to tell. Of the participants, 33% had a vocational degree education, 54% had a bachelor’s degree, and 13% had at least a master’s degree education. The majority of the participants were registered nurses (66%) and licenced practical nurses (19%), and the minority represented, e.g., dental and diagnostics nurses. The sample included public sector workers (80%) and private sector workers (17%), and 3% was employed in the third sector. The most common workplace was hospital (56%), primary care setting (18%) and older people services (15%). Most participants (86%) had one employer, and 14% had two or more employers. The majority (88%) had a permanent employment contract when 12% had a temporary contract.

First, we analysed the correlations between the dimensions of low employment precariousness to identify any excessively high correlations that would need to be accounted for in subsequent analyses. The correlations ranged from *r* = 0.02/0.03 to 0.57/0.57 in 2023/2025, indicating that the dimensions were not too strongly correlated. The CFA showed that, within the eight‐factor model of low employment precariousness, some factor loadings were relatively small (< 0.30). Nevertheless, the overall model demonstrated an excellent fit to the data in both years. In 2023, RMSEA < 0.001 (95% CI 0.000–0.030), CFI = 1.000, and TLI = 1.000. In 2025, model fit indices were RMSEA < 0.001 (95% CI 0.000–0.014), CFI = 1.000 and TLI = 1.000.

The dimensions of employment contract and rights had the highest mean scores in both study years (Table 1). Over the 2‐year period, all dimensions of low employment precariousness showed a significant increase, with the exception of low vulnerability, for which no significant change was observed. The effect sizes for all observed differences were small.

**TABLE 1:** Comparison of study variables and standardized factor loadings by year.

	Mean (SD) 2023	Mean (SD) 2025	*p*	Partial *η* ^2^	Std. loading 2023	Std. loading 2025
Low employment precariousness (scale 0–1^∗^)	0.71 (0.13)	0.74 (0.13)	< 0.001	0.06		
Employment contract	0.88 (0.32)	0.91 (0.28)	0.002	0.01	0.13	0.16
Empowerment	0.73 (0.17)	0.75 (0.15)	< 0.001	0.01	0.36	0.49
Low vulnerability	0.76 (0.18)	0.77 (0.18)	0.060	0.00	0.60	0.73
Rights	0.88 (0.16)	0.90 (0.15)	< 0.001	0.02	0.42	0.26
Enforceability of rights	0.76 (0.18)	0.78 (0.18)	< 0.001	0.01	0.74	0.79
Working times	0.68 (0.18)	0.70 (0.17)	< 0.001	0.05	0.45	0.34
Wages	0.47 (0.50)	0.50 (0.50)	< 0.001	0.02	0.15	0.14
Training	0.59 (0.49)	0.65 (0.48)	0.001	0.01	0.28	0.34
Mental well‐being (scale 1–6)	3.88 (0.91)	3.98 (0.93)	< 0.001	0.01		
Burnout (scale 1–5)	2.56 (0.66)	2.49 (0.63)	< 0.001	0.02		
Job resources (scale 1–4)	3.28 (0.44)	3.29 (0.45)	0.408	0.00		
Psychological safety (scale 1–5)	3.50 (0.77)	3.55 (0.72)	0.042	0.00		
Social support (scale 1–4)	2.95 (0.60)	3.02 (0.60)	< 0.001	0.01		
Work engagement (scale 1–7)	5.43 (1.37)	5.43 (1.36)	0.844	0.00		

Abbreviation: SD = standard deviation.

^∗^0 = highest precariousness, and 1 = lowest precariousness. Std = standardized.

Correlations between the study variables were mostly moderate to strong (Table 2), and all were statistically significant. Strong associations were observed between low employment precariousness in 2023 and low employment precariousness in 2025 (*r* = 0.563), mental well‐being and burnout (*r* = −0.557), mental well‐being and work engagement (*r* = 0.592), burnout and work engagement (*r* = −0.626), and psychological safety and work engagement (*r* = 0.577).

**TABLE 2:** Correlations between the study variables.

	LEP 2023	LEP 2025	MWB 2025	BO 2025	JR 2025	PS 2025	SS 2025	WE 2025
LEP 2023	1							
LEP 2025	0.563^∗∗^	1						
MWB 2025	0.246^∗∗^	0.346^∗∗^	1					
BO 2025	−0.173^∗∗^	−0.304^∗∗^	−0.557^∗∗^	1				
JR 2025	0.301^∗∗^	0.356^∗∗^	0.294^∗∗^	−0.248^∗∗^	1			
PS 2025	0.333^∗∗^	0.452^∗∗^	0.343^∗∗^	−0.399^∗∗^	0.402^∗∗^	1		
SS 2025	0.294^∗∗^	0.419^∗∗^	0.399^∗∗^	−0.434^∗∗^	0.389^∗∗^	0.577^∗∗^	1	
WE 2025	0.237^∗∗^	0.379^∗∗^	0.592^∗∗^	−0.626^∗∗^	0.431^∗∗^	0.401^∗∗^	0.419^∗∗^	1

Abbreviations: BO = burnout, JR = job resources, LEP = low employment precariousness, MWB = mental well‐being, PS = psychological safety, SS = social support, WE = work engagement.

^∗∗^ *p* < 0.01.

The first hypothesis was supported, with the model demonstrating an excellent fit: RMSEA < 0.001 (95% CI 0.000–0.032), CFI = 1.000 and TLI = 1.000. Figure 3 illustrates the autoregressive (from low employment precariousness in 2023 to low employment precariousness in 2025 [*β* = 0.56]), cross‐sectional (from low employment precariousness in 2025 to burnout in 2025 [*β* = −0.30] and mental well‐being in 2025 [*β* = 0.31]), and cross‐lagged (from low employment precariousness in 2023 to mental well‐being in 2025 [*β* = 0.07]) effects. Additionally, although not shown in the figure, mental well‐being and burnout were strongly correlated (*r* = −0.51). The total effect of low employment precariousness in 2023 on mental well‐being in 2025 was *β* = 0.24 (*p* < 0.001), and on burnout *β* = −0.17 (*p* < 0.001).

**FIGURE 3:**
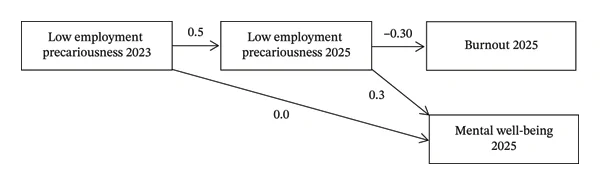
The effects of low employment precariousness on burnout and mental well‐being.

Next, we incorporated the mediators into the model as hypothesized (Model 1, Table 3) including autoregressive path from low employment precariousness in 2023 to low employment precariousness in 2025. In 2025, cross‐sectional paths were specified from low employment precariousness to job resources, psychological safety, social support and work engagement and from these variables to mental well‐being and burnout. The model fit became acceptable by removing the nonsignificant paths from job resources to mental well‐being and burnout and from psychological safety to mental well‐being. We run two additional models guided by modification indices and theoretical plausibility (Table 3). Compared with Model 1, Model 2 demonstrated improvement in model fit (Δχ^2^(1) = 12.01, *p* < 0.001; ΔCFI = 0.004; ΔTLI = 0.008; ΔRMSEA = −0.008). Compared with Model 2, Model 3 demonstrated further improvement in model fit (Δχ^2^(3) = 23.43, *p* < 0.001; ΔCFI = 0.011; ΔTLI = 0.031; ΔRMSEA = −0.042).

**TABLE 3:** Path models testing the mediations.

Model	*χ* ^2^ (df)	CFI	TLI	RMSEA	SRMR	AIC	BIC
1	41.7 (10)	0.989	0.969	0.056	0.032	8097	8254
2	29.7 (9)	0.993	0.977	0.048	0.026	8087	8249
3	6.2 (6)	1.000	1.000	0.006	0.009	8070	8247

*Note:* Model 1 = hypothesized model without nonsignificant paths from job resources (2025) and psychological safety (2025) to mental well‐being (2025). Model 2 = Model 1 + path from low employment precariousness (2025) to mental well‐being (2025). Model 3 = Model 2 + paths from low employment precariousness (2023) to job resources (2025), psychological safety (2025) and social support (2025).

The second hypothesis was partially supported (Figure 4). Low employment precariousness in 2023 showed an autoregressive effect on low employment precariousness in 2025 (*β* = 0.56). Cross‐sectionally, low employment precariousness in 2025 had positive effects on job resources (*β* = 0.28), psychological safety (*β* = 0.40), social support (*β* = 0.38) and work engagement (*β* = 0.38). Furthermore, psychological safety negatively affected burnout (*β* = −0.10); social support positively influenced mental well‐being (*β* = 0.15) and negatively affected burnout (*β* = −0.14); and work engagement positively influenced mental well‐being (*β* = 0.49) and negatively affected burnout (*β* = −0.52). In addition, low employment precariousness in 2025 had a direct positive effect on mental well‐being (*β* = 0.10). Moreover, the total effect of low employment precariousness in 2025 on mental well‐being was *β* = 0.34, and indirect effect on burnout *β* = −0.29. The second hypothesis was only partially confirmed, as job resources had no significant effects on mental well‐being or burnout, and psychological safety did not affect mental well‐being. Additionally, some cross‐over effects were detected. Low employment precariousness in 2023 had significant positive direct effect on job resources (*β* = 0.14), psychological safety (*β* = 0.11) and social support (*β* = 0.08) in 2025. The indirect effect of low employment precariousness in 2023 on mental well‐being in 2025 was *β* = 0.20, and the indirect effect on burnout was *β* = −0.19 (Table 4).

**FIGURE 4:**
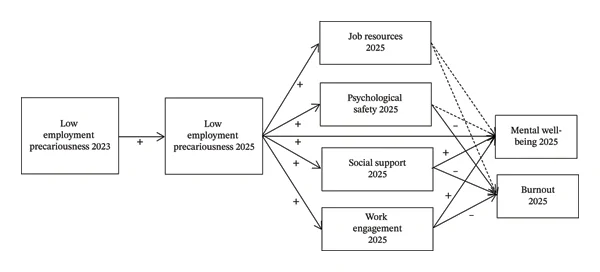
The effects of low employment precariousness on burnout and mental well‐being mediated by job resources, psychological safety, social support, and work engagement. Dashed lines are non‐significant.

**TABLE 4:** Direct, indirect and total effects in the mediation model (*p* ≤ 0.05).

	Direct effect	Indirect effect	Total effect
	*β* (SE)	*β* (SE)	*β* (SE)
LEP23 ⟶ LEP25	0.56 (0.03)		
LEP23 ⟶ JR25	0.14 (0.11)	0.16 (0.07)	0.29 (0.10)
LEP23 ⟶ PS25	0.11 (0.18)	0.22 (0.12)	0.33 (0.16)
LEP23 ⟶ SS25	0.08 (0.15)	0.21 (0.10)	0.29 (0.13)
LEP23 ⟶ WE25		0.21 (0.20)	
LEP23 ⟶ MWB25		0.20 (0.13)	
LEP23 ⟶ BO25		−0.19 (0.08)	
LEP25 ⟶ JR25	0.28 (0.12)		
LEP25 ⟶ PS25	0.40 (0.19)		
LEP25 ⟶ SS25	0.38 (0.16)		
LEP25 ⟶ WE25	0.38 (0.31)		
LEP25 ⟶ MWB25	0.10 (0.20)	0.24 (0.15)	0.34 (0.22)
LEP25 ⟶ BO25		−0.29 (0.11)	
PS25 ⟶ BO25	−0.10 (0.03)		
SS25 ⟶ MWB25	0.15 (0.04)		
SS25 ⟶ BO25	−0.14 (0.03)		
WE25 ⟶ MWB25	0.49 (0.02)		
WE25 ⟶ BO25	−0.52 (0.01)		

Abbreviations: BO = burnout, JR = job resources, LEP = low employment precariousness, MWB = mental well‐being, PS = psychological safety, SS = social support, WE = work engagement.

## 4. Discussion

The study examined the effects of low employment precariousness on mental well‐being and burnout, and the mediating roles of psychosocial working conditions in this relationship. The study produced novel evidence by showing that low employment precariousness associates with better mental well‐being and lower burnout over time among nurses. In the extended mediation model, low employment precariousness was directly associated with better mental well‐being and indirectly associated with mental well‐being through psychosocial pathways. Work engagement and social support emerged as potential mediators, suggesting possible pathways linking low employment precariousness to higher mental well‐being and lower burnout.

Our longitudinal findings align with meta‐analytic evidence showing that high employment quality is positively associated with worker well‐being [37], and lower burnout and greater engagement among nurses [38]. Consistent with the JD‐R theory, psychological and social resources (e.g., engagement and social support) appear to be key pathways through which low employment precariousness translates into mental health benefits [15]. Within healthcare, social support from peers and supervisors has repeatedly been shown to buffer stress and burnout [25], a pattern shown in our mediation results where social support associated with higher well‐being and lower burnout.

Psychological safety related only to lower burnout, and job resources did not relate to either outcome, partially confirming our mediation hypothesis. Psychological safety was associated with lower burnout but not with mental well‐being, consistent with evidence that psychologically safe climates may reduce emotional exhaustion and support adaptation during uncertainty rather than directly enhance well‐being [22]. Alternatively, psychological safety may function primarily as a buffer against negative occupational experiences, whereas positive mental well‐being may depend more strongly on other factors, such as meaningful work, personal resources or conditions outside the workplace. The null findings for job resources align with earlier results that voice or skill‐focused resources can be insufficient to alter burnout when broader structural or relational conditions are adverse [39]. Together, these results underscore that structural employment conditions (low employment precariousness) and relational climates (support and safety) may be more proximal determinants of burnout and well‐being than task‐level resources alone.

Descriptively, most low employment precariousness dimensions increased slightly between 2023 and 2025, with no change in the low vulnerability dimension, suggesting dimension‐specific trajectories in employment quality. In this study, low employment precariousness included contract security, rights, representation, wages, working time, training and management practices. When these conditions improve, workers gain predictability, fairness and protection, which can enable engagement and activate social support networks. The direct effect of low employment precariousness on mental well‐being suggests that high employment quality contributes to general positive functioning beyond its indirect effects via engagement and support. Within JD‐R, low employment precariousness can be conceptualized as a higher order contextual resource: It reduces demands (e.g., job insecurity and unfair scheduling) and enhances resources (e.g., rights and training), creating conditions for sustained vigour and dedication to work [15].

The dimension‐specific stagnation in low vulnerability may indicate organizational leadership practices that lag behind improvements in contractual or rights‐based aspects, which could attenuate the full benefits of low employment precariousness on psychological safety and day‐to‐day support. Management development emerges as a critical priority, as the evidence demonstrates its influence on employees’ well‐being and mental health. Interventions thus need to pair structural improvements with leadership development and work‐community practices to consolidate gains in well‐being [40]. Moreover, management training should emphasize actions at the work community level, strengthening and acknowledging social support. Management practices across organizations should evolve toward open dialogue and a psychologically safe culture of communication.

Our findings support policy investments in increasing high‐quality employment by including secure contracts, enforceable rights, fair wages, predictable working time and access to training as primary prevention for mental health problems and burnout in healthcare. Organizational programs should bolster work engagement (e.g., job crafting, autonomy and feedback) and strengthen social support (peer‐support structures and supervisory coaching). Aligning structural (low employment precariousness) and relational (support and safety) strategies offer a coherent pathway to workforce sustainability, as structural factors might influence individual outcomes, challenging narratives that place the burden of resilience solely on employees. This offers a meaningful contribution to current debates on workforce sustainability. Many nurses who exit their fields self‐blame for lacking resilience, yet systemic conditions, such as precarious contracts, inequitable pay and absent protections, play a critical role. In financially austere times, there is a risk of shifting responsibility onto individuals rather than addressing structural deficiencies.

### 4.1. Strengths and Limitations

A major strength is the prospective two‐wave design with validated instruments (WHO‐5, BAT‐4 and UWES‐9; EPRES‐BE), allowing autoregressive and cross‐lagged modelling of effects across time. Internal consistencies were acceptable to excellent across constructs, and model fit indices were strong, supporting measurement quality. The large baseline sample and trade‐union recruitment improve ecological validity for public‐sector healthcare contexts.

Limitations include self‐reported measures that may introduce common‐method bias, attrition between waves with follow‐up restricted to volunteers (potential selection effects) and listwise deletion, although Little’s MCAR test supported the approach, which can reduce statistical power. We tested the possible nonresponse bias by comparing low employment precariousness, mental well‐being and burnout between the participants who volunteered to follow‐up with those who did not. No significant differences were found. However, the dropout analysis revealed some differences in sociodemographic characteristics. Compared with participants who remained in the study, those who dropped out were more likely to be women, had longer work experience and had lower levels of education. Overall, the follow‐up rate of 57% was acceptable for a longitudinal study. Although mental well‐being and burnout were measured at baseline and controlled for in the analyses, changes in these outcomes over time were not directly examined. Accordingly, the findings indicate longitudinal associations rather than trajectories or changes in mental well‐being and burnout across the follow‐up period. Finally, generalizability beyond Finnish nurses and union‐connected populations should be undertaken cautiously.

## 5. Conclusions

Low employment precariousness associates with better mental well‐being and lower burnout among nurses over time. Work engagement and social support act as key mediators, while psychological safety serves as a protective factor against burnout. Beyond individual resilience, structural employment conditions and supportive organizational climates are likely to play a key role in promoting better mental health in healthcare. Organizations should prioritize leadership quality and supportive management practices, as these seem to be crucial for enhancing mental well‐being and reducing burnout. Future research should further explore dimension‐specific trajectories (particularly in management) and create multilevel interventions that integrate policy‐level improvements with unit‐level leadership and support practices.

## Funding

This work was supported by the Finnish Work Environment Fund (grant number 230355).

## Conflicts of Interest

The authors declare no conflicts of interest.

## Supporting Information

Additional supporting information can be found online in the Supporting Information section.

## Supporting information


**Supporting Information** STROBE statement—checklist of items that should be included in reports of cohort studies.

## Data Availability

Data are available from the first author upon reasonable request.
